# Multidisciplinary Management of Foreign Body Aspiration in Pediatrics: A Case Complicated by Bilateral Pneumothorax and Respiratory Failure

**DOI:** 10.7759/cureus.78287

**Published:** 2025-01-31

**Authors:** Huda Mohammad Zarie, Hawazen Yousef Alani, Ahmad A Bukhari, Hazem Ibrahim Shokry, Mansour Rabeh Alshamani

**Affiliations:** 1 Pediatric Critical Care, Ohud Hospital, Madinah, SAU

**Keywords:** bilateral pneumothorax, extracorporeal membrane oxygenation (ecmo), foreign body aspiration, multidisciplinary management, pediatric respiratory distress

## Abstract

Foreign body aspiration (FBA) is a significant cause of morbidity and mortality in the pediatric population, particularly in children under three years of age. This condition often presents with nonspecific respiratory symptoms, leading to delays in diagnosis and an increased risk of severe complications such as pneumothorax and respiratory failure. Here, we present a case of a 2-year-old child with FBA complicated by bilateral pneumothorax, severe respiratory acidosis, and left lung collapse. Diagnostic imaging, including computed tomography (CT), identified an obstruction in the left main bronchus. High-frequency oscillatory ventilation (HFOV) and multidisciplinary planning were critical in stabilizing the patient before definitive management with rigid bronchoscopy under high-risk conditions. The case highlights the diagnostic and therapeutic challenges associated with severe FBA and underscores the importance of advanced therapeutic modalities, such as extracorporeal membrane oxygenation (ECMO), in managing critically ill pediatric patients.

## Introduction

Foreign body aspiration (FBA) is a critical condition in pediatrics, accounting for a significant number of emergency visits globally. It is particularly prevalent in children under three years of age, where developmental curiosity and immature protective airway reflexes increase the risk of aspiration [1]. FBA is associated with high morbidity and, in severe cases, mortality, necessitating prompt diagnosis and intervention [2].

The clinical presentation of FBA varies widely, often mimicking common respiratory conditions such as asthma or pneumonia, leading to potential delays in diagnosis [3]. While chest radiographs are typically the first-line imaging modality, they may fail to detect radiolucent foreign bodies, emphasizing the importance of advanced diagnostic tools like computed tomography (CT) [4].

Management of FBA requires a multidisciplinary approach, with rigid bronchoscopy under general anesthesia being the gold standard for foreign body removal. However, in cases complicated by respiratory distress or failure, such as in this report, additional interventions like high-frequency oscillatory ventilation (HFOV) and extracorporeal membrane oxygenation (ECMO) may be considered [5,6].

This case highlights the challenges of managing a complex presentation of FBA in a pediatric patient with severe complications, including bilateral pneumothorax and acute respiratory acidosis, requiring innovative therapeutic strategies and collaborative care.

## Case presentation

A 2-year-old Egyptian boy, previously healthy and fully vaccinated, traveled to Saudi Arabia for Umrah. He presented with a history of cough and fever for three days, accompanied by decreased feeding and shortness of breath for one day. He was admitted to a Ministry of Health hospital with type 2 respiratory failure, requiring intubation and mechanical ventilation.

**Figure 1:**
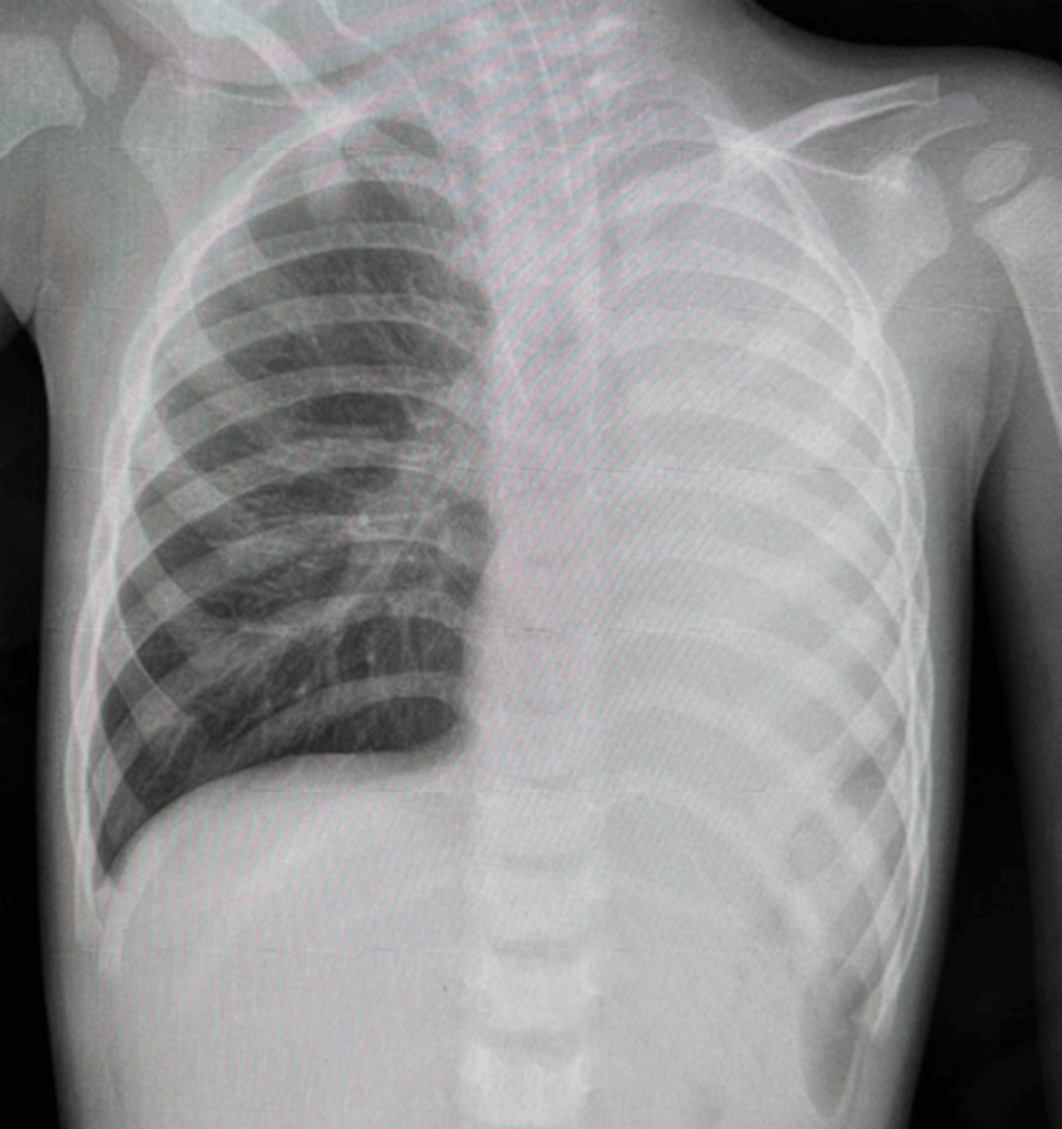
Antero-posterior chest X-ray showing total left lung collapse with three white arrows demonstrating the collapse.

Initial chest X-ray showed total left lung collapse, prompting a CT scan that revealed an intraluminal hypodensity in the left mainstem bronchus, likely due to inspissated secretions or a foreign body, causing complete left lung collapse (Figure 1). The right lower lobe demonstrated patchy ground-glass opacities and basal consolidative changes. A mucus plug or foreign body was suspected, and ENT consultation for rigid bronchoscopy was recommended.

**Figure 2:**
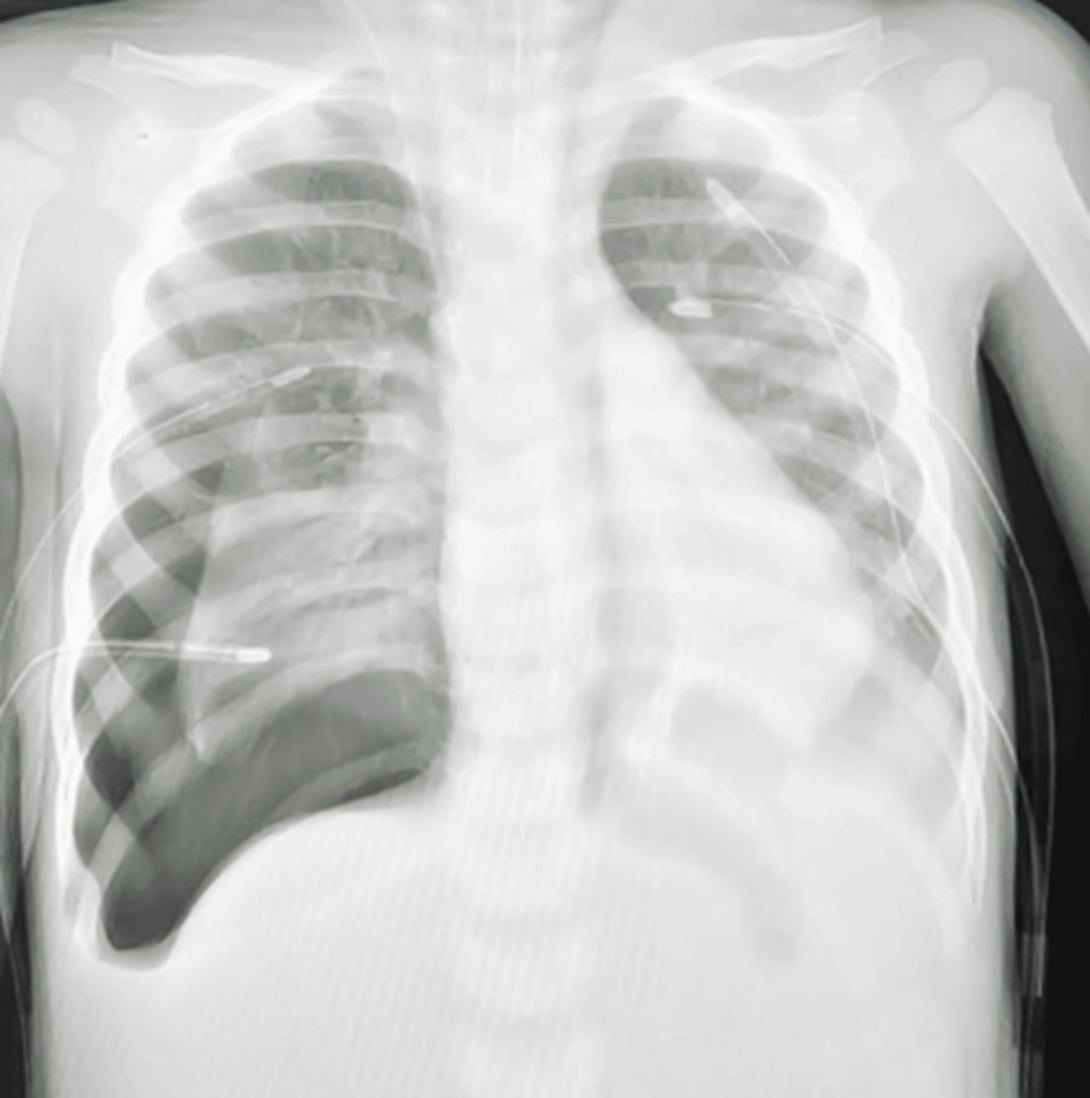
Antero-posterior chest X-ray showing bilateral pneumothorax.

Transferred to Ohud Hospital, the patient’s condition worsened with hypercapnia and hypoxia, necessitating high-frequency ventilation. Blood gas analysis showed persistent respiratory acidosis with a pH of 7.10-7.25 and PaCO2 levels of 80-100 mmHg. A respiratory viral panel detected influenza A, complicated by acute respiratory distress syndrome (ARDS) and bilateral pneumothorax, which required chest drains (Figure 2). Bedside rigid bronchoscopy revealed granulation tissue obstructing the left main bronchus, but removal attempts were limited by the patient’s instability.

Despite escalating ventilatory support, including HFOV, the patient’s condition progressed to severe respiratory acidosis by day eight (pH 6.80, PaCO2 180 mmHg) with an oxygen saturation index of 30. After multidisciplinary discussions and obtaining parental consent, a high-risk bronchoscopy was performed in the operating room. While veno-venous extracorporeal membrane oxygenation (V-V ECMO) was considered as an alternative, it was ultimately declined due to its associated complications.

**Figure 3:**
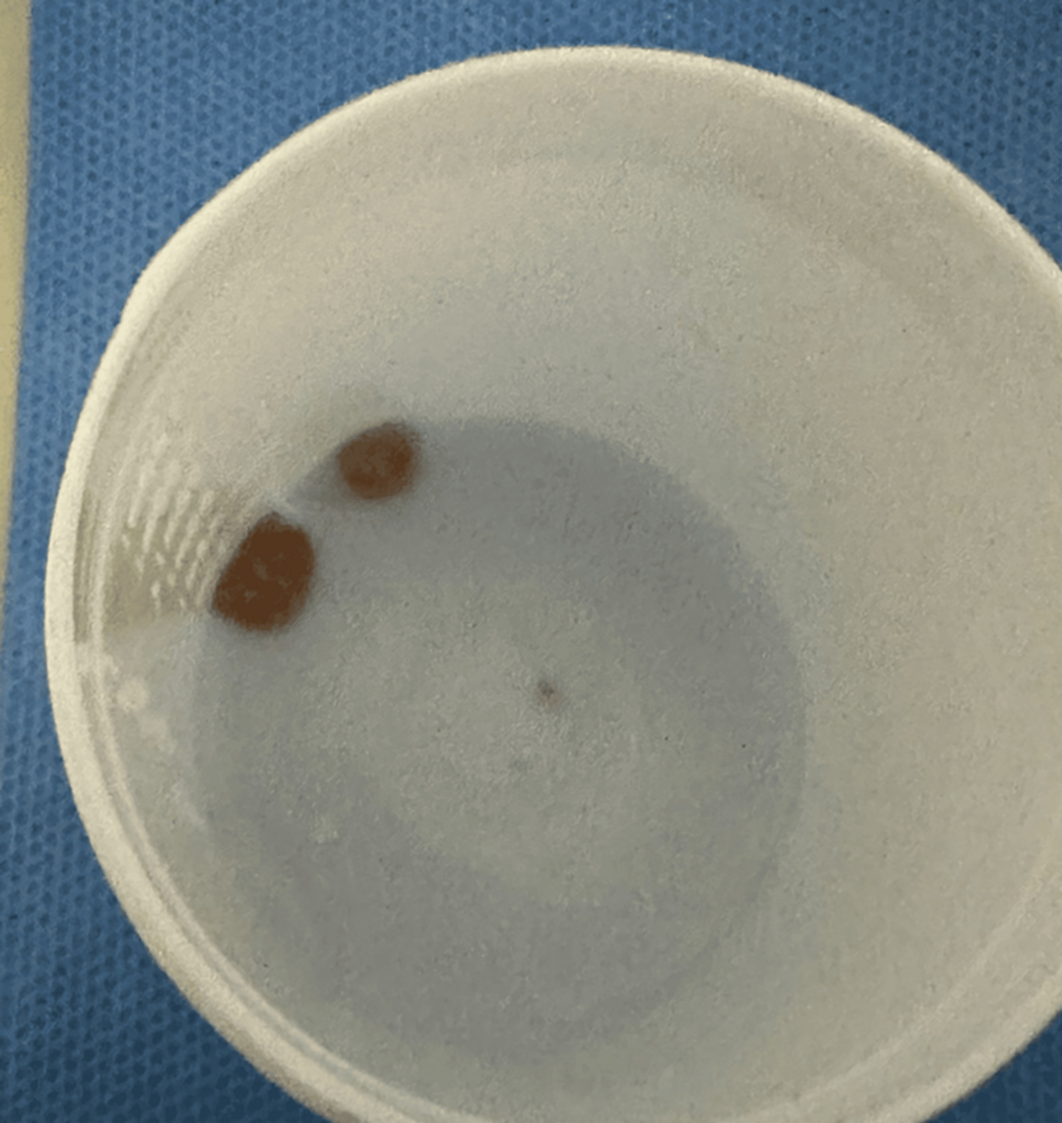
Postoperative image showing foreign body fragments removed from the left main bronchus.

Under general anesthesia in the operating room, telescopic examination revealed granulation tissue and foreign body fragments in the left main bronchus (Figure 3). These were successfully removed, with bleeding controlled using adrenaline irrigation. Post-procedure, the patient was stabilized and transferred to the pediatric intensive care unit.

**Figure 4:**
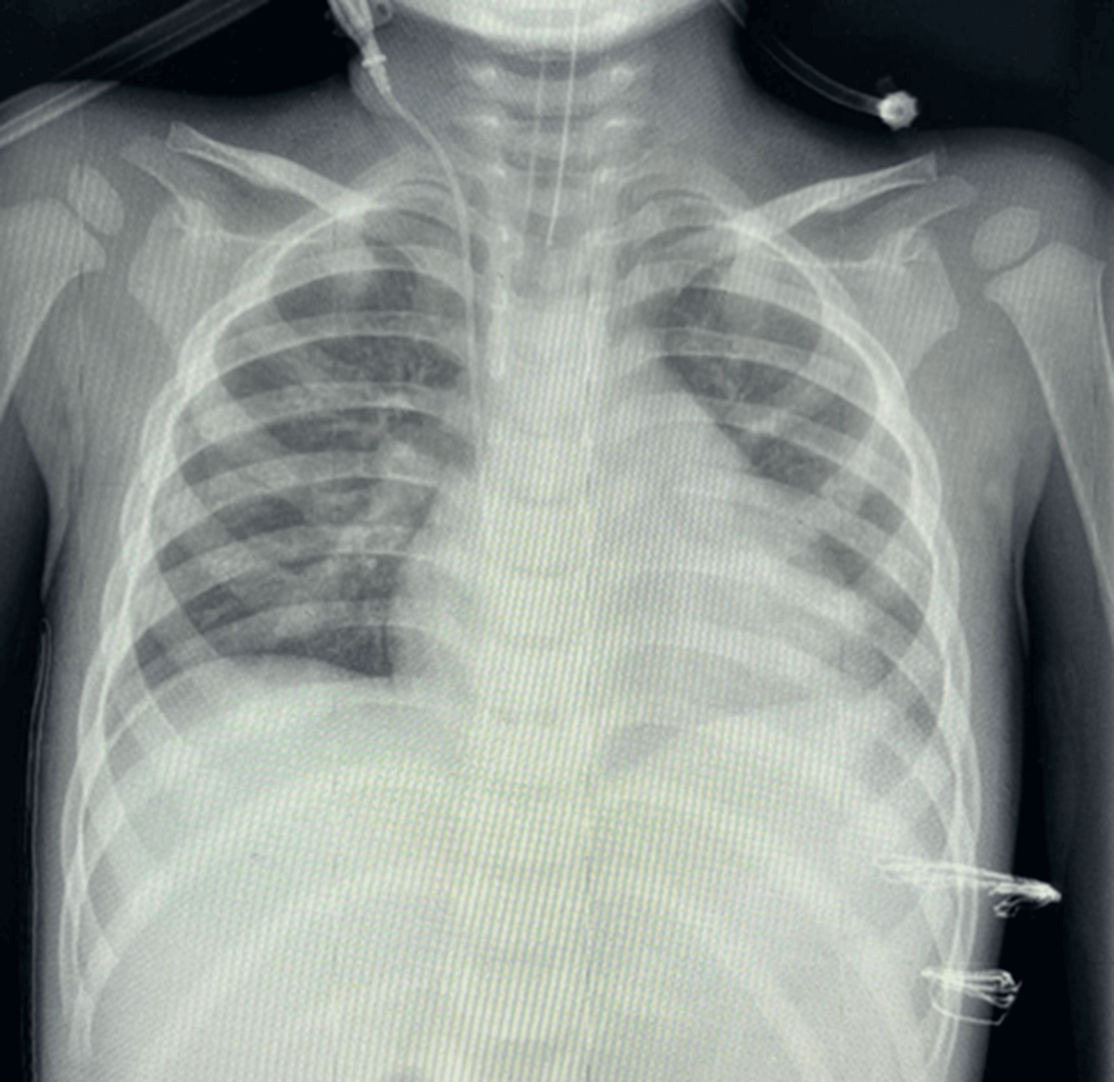
Antero-posterior chest X-ray taken after the removal of the foreign body and chest drain, with the patient still intubated.

Over the following week, the patient’s condition improved. Bilateral pneumothorax was resolved, chest drains were removed, and he was successfully extubated to a nasal cannula. He was discharged after completing oxygen weaning, sedation withdrawal, and antimicrobial therapy (Figure 4).

## Discussion

FBA is a common and potentially life-threatening condition in pediatrics, predominantly affecting children under three years of age. Typical causative agents include organic materials like nuts, seeds, and small objects. The incidence varies globally but remains a significant public health issue, with an estimated rate of 20.4 emergency visits per 100,000 children annually [1,2]. Despite its frequency, diagnosis is often delayed due to nonspecific symptoms and the absence of a witnessed aspiration event.

Children with FBA commonly present with coughing, wheezing, and dyspnea, but atypical presentations can mimic asthma or respiratory infections. Imaging plays a crucial role, yet chest X-rays may fail to detect radiolucent foreign bodies, underscoring the importance of a high index of suspicion [3]. Advanced imaging modalities, such as CTs, are invaluable in identifying obstructions and associated complications, aiding in timely diagnosis and management [4].

The gold standard for FBA management is bronchoscopic removal under general anesthesia [5]. However, this intervention poses challenges in patients with severe respiratory compromise. Effective management requires a multidisciplinary approach, including stabilization through advanced ventilatory strategies like HFOV before proceeding with bronchoscopy [6,7]. This careful orchestration minimizes perioperative risks and maximizes the likelihood of a successful outcome.

In our patient, the clinical course was complicated by severe respiratory acidosis and bilateral pneumothorax, requiring intensive ventilatory support and meticulous planning for intervention. Initial imaging revealed complete left lung collapse and a left main bronchus obstruction. Despite high-frequency ventilation and other supportive measures, the patient remained unstable, prompting the decision to perform rigid bronchoscopy under high-risk conditions. This procedure successfully removed multiple foreign body fragments and alleviated the obstruction. The resolution of complications and subsequent recovery highlight the importance of timely, multidisciplinary interventions in managing such critical cases.

ECMO has emerged as a life-saving modality in cases of refractory respiratory failure [8]. While its use in FBA is rare, ECMO provides critical support by stabilizing gas exchange and hemodynamics, allowing safer intervention. Literature highlights its role as a bridge to recovery in critically ill pediatric patients, underscoring its expanding applications in high-risk airway cases [9,10]. Decisions to implement ECMO must consider the balance of risks, parental consent, and institutional expertise [11].

FBA is associated with a range of complications, including pneumonia, atelectasis, and pneumothorax [2]. Prompt recognition and timely intervention are pivotal in reducing morbidity. Despite the complexities of management, prognosis in appropriately managed cases remains favorable. Long-term outcomes are closely linked to the resolution of acute complications and the absence of significant delays in treatment [1,12].

## Conclusions

FBA remains a critical condition in pediatric care, demanding prompt diagnosis and tailored intervention strategies. This case underscores the importance of advanced imaging and multidisciplinary collaboration in addressing complications such as pneumothorax and respiratory acidosis. The integration of life-saving techniques like ECMO further highlights the evolving capabilities of modern medicine in managing severe cases. Ultimately, early recognition, timely management, and the use of innovative therapeutic approaches are essential for improving patient outcomes and reducing associated morbidity and mortality.
